# A Brief Educational Intervention to Improve Healthcare Providers' Awareness of Child Passenger Safety

**DOI:** 10.1155/2013/821693

**Published:** 2013-02-11

**Authors:** O. James Ekundayo, Gennifer Jones, Angela Brown, Muktar Aliyu, Robert Levine, Irwin Goldzweig

**Affiliations:** ^1^Department of Family and Community Medicine, Meharry Medical College, 1005 Dr. D. B. Todd Jr. Boulevard, Nashville, TN 37208, USA; ^2^Department of Preventive Medicine, Vanderbilt University, Suite 750, 2525 West End Avenue, Nashville, TN 37203, USA

## Abstract

*Introduction*. Motor vehicle crashes are the leading cause of death among US children aged 4–14 years. In theory, health provider counseling about Child Passenger Safety (CPS) could be a useful deterrent. The data about the effectiveness of CPS dissemination is sparse, but existing results suggest that providers are not well informed. Moreover, there is insufficient evidence to determine whether provider counseling about CPS is effective. *Methods*. We therefore assessed CPS best practice knowledge among 217 healthcare workers at hospitals in seven cities throughout the USA and evaluated the impact of a brief, lunch and learn educational intervention with a five-item questionnaire. Attendees were comprised of physicians, nurses, social workers, pediatric residents, and pediatric trauma response teams. *Results*. Pre-post survey completion was nearly 100% (216 of 217 attendees). Participation was fairly evenly distributed according to age (18–29, 30–44, and 45+ years). More than 80% of attendees were women. Before intervention, only 4% of respondents (9/216) answered all five questions correctly; this rose to 77% (167/216) (*P* < 0.001, using a Wilcoxon signed-rank test) after intervention. *Conclusion*. Future research should consider implementation and controlled testing of comparable educational programs to determine if they improve dissemination of CPS best practice recommendations in the long term.

## 1. Introduction

Unintentional injuries due to motor vehicle crashes (MVCs) are the leading cause of death and long-term disability between the ages of 4–14 years [1, 2]. When properly installed, child safety seats have been shown to reduce the risk of childhood injury by 71% to 82% and death by 28% relative to seat belts alone [3–6]. Within the subset of four- to eight-year olds, booster seats reduced the risk of nonfatal injury by 45% relative to seat belts alone [7]. Nonetheless, about 50% of the 1,500 US children who die in MVCs each year are unrestrained [8]. 

The American Academy of Pediatrics (AAP) and the National Highway Traffic Safety Administration (NHTSA) and many others promote the importance of child restraint in vehicles [9, 10]. Moreover, for more than a decade, the AAP has published best practice clinical algorithms as an aid to promoting restraint use by health care providers. The failure of such approaches to effectively translate research knowledge into practice is reflected in the US Preventive Services Task Force finding of insufficient evidence to support an incremental benefit from provider counseling about motor vehicle occupant restraint independent from legislation and community-based interventions [11], the failure to identify child passenger safety (CPS) as a cost-effective clinical preventive service for the general population, [12] and low-frequency age-appropriate child restraint use for the nation with racial and ethnic disparities in the use of age-appropriate child restraints clearly evident [13]. 

In theory, clinical encounters provide a good opportunity to educate families/caregivers and influence behavior change [13]. Such encounters may have little chance of success, however, if providers are unaware of best practice recommendations. While data is sparse about specific knowledge of best practices, the information which does exist gives cause for concern. Among pediatric emergency physicians, for example, only 36% correctly answered all questions about the AAP/NHTSA CPS recommendations [14]. The reasons given for lack of knowledge include inadequate curriculum time [15], an explanation which is buttressed by a survey of 390 emergency medicine graduate physicians in training, 62% of whom reported having had little injury prevention training. Only 44% reported receiving lectures on injury prevention as a resident, and only 28% reported that they consistently read journal articles on the subject [15]. The present program therefore sought to assess provider knowledge of CPS best practice recommendations and to evaluate a brief educational intervention.

## 2. Methods

### 2.1. Academic-Business-Community Alliances

We have previously reported promising results in the area of automobile safety based, in part, on identification of issues where the interests of academic institutions, for-profit businesses, and communities intersect [16]. The present project—see me safe (SMS)—is an extension of that model and includes a collaboration between Meharry Medical College, the Ford Motor Company Fund, community-based organizations, including elementary schools, and providers from pediatric facilities in hospitals in seven cities throughout the nation which had been identified as having an interest in CPS based on information from CPS experts, referrals from injury prevention colleagues, and our previous experiences with some of these facilities. 

 The primary aim of SMS is to increase the capacity of community-based organizations and core teams of CPS stakeholders to increase services by helping them to develop supportive networks including physicians, nurses, social workers, pediatric residents, and pediatric trauma response teams through a Prescription for Safety (PFS) education program. The PFS curriculum is designed to assist healthcare providers to increase knowledge about CPS best practice recommendations enabling them to counsel parents and caregivers about the importance of proper use of safety restraint systems for their children and make appropriate referrals to certified CPS technicians, consistent with best practice recommendations [9].

### 2.2. Program

Fliers and other announcements inviting participation in the education program were sent to pediatricians, residents, nurses, and other healthcare professionals involved in the care of children. Because of established disparities in automobile restraint use among minority populations [13], healthcare facilities where culturally diverse populations, especially African American and Hispanic families receive care, were specifically targeted. The PFS workshops were arranged to fit into the ongoing continuing education/training schedules at each facility.

The PFS education intervention was a “lunch and learn” education program. The curriculum was designed by Meharry faculty and staff based on a thorough literature review and recommendations from the AAP, standards set by NHTSA, and information from the Insurance Institute for Highway Safety [9, 10]. The curriculum covered (a) information on the leading causes of death for children aged 4–14; (b) the aforementioned CPS guidelines set forth by the AAP and NHTSA; (c) available local and national resources that promote proper child restraint use; and (d) the need to promote best practices in pediatric health care as it relates to CPS and the referral of parents to a nationally certified CPS technician for education and guidance about CPS. The curriculum emphasized two basic steps for CPS. First, all child passengers in motor vehicles should ride in rear seat until age 13. Second, all child passengers should be restrained properly in rear-facing child seat, forward-facing child seat, booster seat, or lap-shoulder belt. Evidence in support of these steps culminated in a PFS four-item summary which covered rear-facing seats, forward-facing seats, booster seats, and safety belts. The presentation was delivered by an experienced CPS instructor, certified by Safe Kids Worldwide. This was followed by 20 minutes of questions and answers. An updated version of the presentation is included in the appendix.

 At the training session, participants were given training kits containing a consent form for participation in the pre and post survey, the pre- and postsurvey questionnaires, and a copy of the PowerPoint education presentation. The training kits also included citations of relevant professional publications related to CPS, a handy PFS pad to prescribe a visit to a certified CPS technician to parents/caregivers after consultation, and a single-page summary of current recommendations/standards of care related to CPS. 

### 2.3. Measurements and Outcomes

The survey instrument for this study was developed based on a comprehensive review of the literature on causes of morbidity and mortality in children and current CPS guidelines as recommended by AAP and NHTSA. The survey included multiple choice and free text questions. The main outcome measure was postintervention knowledge of national AAP/NHTSA CPS recommendations among participating healthcare providers. 

### 2.4. Statistical Analysis

Frequency distributions were computed for selected demographic characteristics of the participants. To assess the level of pre- and postintervention knowledge about CPS, overall proportions of participants correctly answering each of the five CPS knowledge questions were calculated. McNemar's chi-square tests were used to determine whether the number of participants correctly answering each question significantly increased between pre- and postintervention assessments. To determine whether there is an overall difference in pre- and postintervention knowledge, the total number of correct answers for each of the five questions was compared between the pretest and the posttest using a Wilcoxon matched-pairs signed-rank test. All tests were two-tailed, and a *P* value of 0.05 or less was considered statistically significant. The data were analyzed using SPSS for Windows release 19 [17].

## 3. Results

Overall, 217 healthcare providers participated in the PFS education sessions across 7 selected cities (Atlanta, Chicago, Miami, Nashville, Phoenix, San Antonio, and San Diego). Of these, 216 completed the pre- and posteducation surveys. More than 80% of the participants were women. Participants were evenly distributed across age groups: 18–29 (30.1%), 30–44 (38.0%), and ≥45 (31.9%). The study population was heterogeneous in terms of racial and ethnic composition. Demographics of the 216 participants who completed the survey are shown in Table 1.

Among all 216 respondents, 89.4% correctly answered the question on the leading cause of death among children (4–14 years) in the United States in the pre-intervention survey. After intervention, 97.2% correctly answered the question (McNemar's chi-square test; *P* = 0.002). On the minimum age at which children should graduate from a rear-facing child safety seat to a forward-facing child safety seat, 63.4% correctly answered the question before intervention compared to 94.0% after intervention (*P* < 0.0001). Also, 68.1% correctly answered the question on when a child should transition to a booster seat before intervention compared to 95.4% after educational intervention (*P* < 0.0001). The proportion of participants who answered the question on the height when children are ready to graduate from a booster seat to wearing lap-shoulder belts was 27.8% before intervention. This number increased to 98.1% after intervention (*P* < 0.0001). Finally, only 15.3% of respondents correctly answered the question, “until what age are children safest riding in the back seat?” prior to the educational intervention, compared to 86.1% after intervention (*P* < 0.0001) (Table 2). Overall, only 9 (4.2%) answered all five questions correctly before intervention compared to 167 (77.3%) after intervention. The brief educational intervention was associated with a significant change in CPS knowledge (Wilcoxon matched pairs, *P* < 0.0001). The median score rating was 3 pre-intervention and 5 postintervention.

In response to our question on whether the training added to participants' knowledge regarding CPS, 171 (79.2%) strongly agreed, 41 (19.0%) agreed, only 1 (0.5%) disagreed, and 3 (1.4%) strongly disagreed. There was no difference in racial/ethnic composition, age, or geographical location between participants with CPS knowledge, both after and before intervention. The questionnaire for the study and the answers to the questions are attached as an appendix.

## 4. Discussion

Preintervention results confirm previous reports about limited provider knowledge of best practice recommendations from the AAP [14]. Postintervention results show that a brief educational program significantly improved knowledge in these participants. Taken together with the existing literature, preintervention results therefore support at least two hypotheses concerning dissemination of knowledge about CPS: (a) publication of best practice recommendations, even by well-respected organizations such as the AAP and NHTSA, is insufficient; and (b) sufficient evidence to support the effectiveness of provider counseling is unlikely to be forthcoming without effective attention to improving provider knowledge. 

Overall, it has been suggested that if states closed all remaining gaps in their child occupant restraint laws and all children (age 0–15) were properly restrained 100 percent of the time, up to 630 additional children's lives would be saved and another 182,000 serious injuries would be prevented every year [18]. Pre-post improvement in the present results supports the additional hypothesis that a brief educational intervention might help to address the problem of proper restraint.

 Many MVCs are preventable and even when they become inevitable, the associated morbidity and mortality can be minimized through the correct use of safety restraints. This is especially true in children (4–14 years old), among whom MVCs have remained the leading cause of death [1, 2]. Although child restraint and seat belt use rates have increased in recent years (from 15% in 1999 to >80% in 2008 for children 0–8), there is still room for improvement [19]. Barriers still remain to the achievement of optimum safety seat use in children. Findings from this study suggest that healthcare professionals, through their lack of knowledge, may be contributing to suboptimal use of car safety seats. As a trusted source of information for parents and care-givers, healthcare providers are critical in promoting CPS. It is important that they are current in CPS recommendations.

What we highlighted in our study is beyond just educating a group in masses and showing increased knowledge. Our findings showed that in spite of more than a decade of publication of best practice clinical algorithms as an aid to promoting restraint use by the AAP and NHTSA, knowledge of CPS recommendations remains low among health care workers. While it could be assumed that educating health care providers will improve CPS knowledge, available evidence shows that training curriculum is inadequate. This explanation is supported by a survey of 390 emergency medicine residents of whom 62% reported that inadequate time is dedicated to injury prevention in training. Only 44% reported receiving lectures on injury prevention and only 28% reported that they consistently read journal articles on the subject [15].

While data is sparse about specific knowledge of best practices, the information which does exist gives cause for concern. In a recent publication of study conducted among pediatric emergency physicians, for example, only 36% correctly answered all questions about the AAP/NHTSA CPS recommendations [14]. We believe that the information provided in this study sheds light on the problem of translating research into practice as it applies to CPS and served as the justification for the intervention we propose. 

The intervention to see that this educational session will improve healthcare workers behavior to discuss CPS with families/caregivers is the next phase of this study, but we do not have data to report at this time. We propose a multicenter, controlled trial to determine whether education in CPS increases health professionals' knowledge on the long term and influences practice behavior. We intend to recruit pediatric primary care providers and nurses. The intervention group, apart from receiving the educational training, will also receive a preventive practice reminder to insert in patient charts, which will consist of CPS checklist to discuss with parent/caregivers. Families that are not in compliance with CPS recommendations will be counseled and referred to a trained on-site clinic-based CPS certified technician. The control group will receive the educational intervention only and continue with their standard of care practice. At the end of the intervention period, we will determine whether providers incorporate the new knowledge about CPS into their practice by comparing the intervention group with the control group on whether they counseled families/caregivers about CPS and referred the family to the onsite certified CPS technician. We will also determine whether provider counseling is effective in changing parent/caregiver attitudes and practices, and ultimately, whether this is associated with incremental improvement in burden of injuries that are sustained by children from MVCs, independent from community-based efforts such as laws.

Our study has several limitations. Pre-post testing of participants with an interest in the topic (evidenced by response to an invitation) does not have the rigor of randomized, controlled trials involving more representative population samples. Also, given the relatively low level of knowledge demonstrated in the pretest, we cannot say how much of the attendance was motivated by personal concern about that lack of knowledge. Possibly, systematic and unbiased selection of participants would produce different results. Further, because attendance was voluntary, present observations showing no significant difference in outcomes according to race, ethnicity, age, or geographic location in the present results should also be interpreted with caution. The same caution would have applied to comparisons according to participant education or job title, but that information was not available for analysis. Finally, since the posttest was given immediately after the educational program and no followup is available, we are unable to comment on retention or other long-term impact. 

Despite the limitations, the present data are consistent with a key observation reported by others in the scientific literature—namely, that knowledge of practice-based guidelines about CPS is insufficient. The results also show, however, that even a brief educational intervention may result in significant improvement in health provider knowledge. The data therefore support the potential utility of formal testing to see if brief educational presentations might improve diffusion of future editions of best practice guidelines for CPS.

## Figures and Tables

**Table 1:** Demographic characteristics of participants: the ford see me safe child passenger safety education study.

Variable	*n* (%)
City	
Atlanta	13 (6.0)
Chicago	29 (13.4)
Miami	48 (22.2)
Nashville	30 (13.9)
Phoenix	16 (7.4)
San Antonio	19 (8.8)
San Diego	61 (28.2)
Age group	
18–29	65 (30.1)
30–44	82 (38.0)
45+	69 (31.9)
Sex	
Female	177 (81.9)
Male	36 (16.7)
Unknown	3 (1.4)
Race/ethnic group	
White	77 (35.6)
Black	67 (31.0)
Hispanic	42 (19.4)
Asian	10 (4.6)
American Indian	5 (2.3)
Unknown	15 (6.9)

**Table 2:** Responses to child passenger safety questions before and after educational intervention: the ford see me safe child passenger safety education study.

Questions	before intervention *n* (%)	after intervention *n* (%)	*P* value*
What is the leading cause of death among children (4–14 years) in the USA?			
Correct	193 (89.4)	210 (97.2)	0.002
Incorrect	23 (10.6)	6 (2.8)	

What is the minimum age at which children can graduate from a rear-facing child safety seat to a forward-facing child safety seat?			
Correct	137 (63.4)	210 (94.0)	<0.0001
Incorrect	79 (36.6)	13 (6.0)	

When should a child transition to a booster seat?			
Correct	147 (68.1)	206 (95.4)	<0.0001
Incorrect	69 (31.9)	10 (4.6)	

At about what height are children generally ready to graduate from a booster seat to wearing only a lap-shoulder belt?			
Correct	60 (27.8)	212 (98.1)	<0.0001
Incorrect	156 (72.2)	4 (1.9)	

Until what age are children safest riding in the back seat?			
Correct	33 (15.3)	186 (86.1)	<0.0001
Incorrect	183 (84.7)	30 (13.9)	

*McNemar's chi square test.
